# Editorial: Omics approach to study the biology and virulence of microorganisms causing zoonotic diseases

**DOI:** 10.3389/fmicb.2022.988983

**Published:** 2022-08-01

**Authors:** Mali Salmon-Divon, Yongqun Oliver He, David Kornspan, Zezhang Tom Wen

**Affiliations:** ^1^Department of Molecular Biology and Adelson School of Medicine, Ariel University, Ariel, Israel; ^2^Unit for Laboratory Animal Medicine, University of Michigan, Ann Arbor, MI, United States; ^3^Department of Microbiology and Immunology, University of Michigan Medical School, Ann Arbor, MI, United States; ^4^Center of Computational Medicine and Bioinformatics, University of Michigan, Ann Arbor, MI, United States; ^5^Department of Bacteriology, Kimron Veterinary Institute, Bet Dagan, Israel; ^6^Department of Prosthodontics, School of Dentistry, Louisiana State University Health, New Orleans, LA, United States; ^7^Department of Microbiology, Immunology and Parasitology, School of Medicine, Louisiana State University Health, New Orleans, LA, United States

**Keywords:** zoonotic diseases, omics analysis, virulence, pathogens, host-microbe interaction

Zoonotic diseases, also known as zoonoses, are infections and diseases that can naturally spread between vertebrate animals and humans. Bacteria, viruses, parasites, protozoa, fungi, and other pathogens can cause different zoonosis. Zoonotic diseases represent a major public health problem worldwide. More than three-quarters of human diseases are caused by pathogens originating from animals or from products of animal origin.

With the development of molecular and bioinformatics tools, some significant progresses have been made over the last decades concerning the biology and the virulence of some of these pathogens. However, methods for quick diagnosis and effective infection control including vaccines and therapeutics for some of the important zoonotic diseases are still lacking.

The technologies for generating omics information have substantially progressed in recent years and became available to an increasing number of researchers. Integration of omics information resulted in extensive advances in many aspects of Zoonoses, from microbial detection to delineating mechanisms of pathogenicity and understanding zoonotic disease epidemiology. Omics information includes: Genomics, that is used to study the diversity of the genomes of emerging zoonotic pathogens and the genetic and molecular basis of the host response; Transcriptomics, that allows the detection of transcriptional regulation mechanisms employed by the pathogen and by the response of its host; Proteomics, which may uncover essential interactions between the pathogen and the host cells; Epigenomics, which plays a significant role in bacterial phenotypes that are not encoded in the genome and contribute another level of regulation by which the host cells integrate and respond to pathogens signals; and Metabolomics, that provides a chemical fingerprint of thousands of metabolites present in cells, and sheds new light on the complexity of the host metabolism as well as of the host-pathogen interaction in each stage of the disease. Integration of signals from all these levels of information will contribute to our understanding of the complete system.

This Research Topic includes five articles that illustrate the use of current omics and systems approaches in studying the biology and virulence of zoonotic pathogens; the interactions of these microorganisms with their host; and the development of computational models on microbe-disease association prediction. Below we provide a summary of these five articles.

Kornspan et al. reported the identification of protein biomarkers for discriminating *Brucella melitensis* field isolates from *B. melitensis* Rev. 1 vaccine strain by MALDI-TOF MS technology. Following *in-silico* proteome comparison, 113 proteins were detected as homologous in both *B. melitensis* 16M and *B. melitensis* Rev.1. Of these, 17 potential biomarker pairs bearing a mass within the working range of MALDI-TOF MS. MALDI-TOF MS analysis detected two unique biomarkers with properties most similar to the ribosomal proteins L24 and S12 which clearly discriminated *B. melitensis* Rev.1 from the closely related *B. melitensis* 16M and field isolates. This study demonstrated the using of MALDI-TOF MS technology for strain differentiation and described ribosomal marker proteins for distinguishing the vaccine strain Rev.1 from field strains. This finding may improve the differential diagnosis necessary for brucellosis control efforts within vaccination programs and subsequently the successful eradication of the zoonoses from small ruminants.

Kuleš et al. used combined untargeted and targeted metabolomics approaches to the metabolic profiles in canine babesiosis with different levels of kidney function. Canine babesiosis is a tick-borne disease caused by haemoprotozoan parasites under the genus Babesia. Acute kidney injury is one of the most prevalent complications of Canine babesiosis. Their study included 22 dogs naturally infected with *Babesia canis* and 12 healthy dogs. Their metabolomics analysis identified many features and metabolites with significantly different concentrations between the healthy group and groups of dogs with babesiosis and different levels of kidney function. The kidney dysfunction accompanying canine babesiosis was found to be associated with many urinary changes in areas including amino acid metabolism, energy metabolism, fatty acid metabolism, and different biochemical pathways. Possible urinary biomarkers may be identified from the list for monitoring renal damage in babesiosis.

Cohn et al. used RNA-seq and comparative transcriptomics to characterize *Salmonella enterica* serovars Javiana and Cerro, two understudied non-typhoidal *Salmonella* serovars. Results showed that like *S*. Typhimurium, both *S*. Javiana and *S*. Cerro displayed significantly higher transcript abundances of the core genes than those for the accessory genes. Compared to *S*. Cerro, *S*. Javiana as well as *S*. Typhimurium had significantly higher abundances of the *Salmonella* Pathogenicity Island 1 transcripts, including *hilA* and *sicA*. Among the orthologous clusters identified in higher abundance in *S*. Cerro, a number of genes are involved in metabolic processes. These results provide novel insights concerning how *S*. Cerro and *S*. Javiana may adapt and survive in the distinctive hosts and impact their abilities to cause diseases in others.

Hernández-Cabanyero et al. performed a time series transcriptomic study to investigate the mechanism by which the zoonotic pathogen *Vibrio vulnificus* triggers death of their fish host. They used the eel immersion infection model and analyzed the transcriptome of red blood cells (RBCs), white blood cells (WBCs) and whole blood using eel-specific microarray platform. They revealed that *V. vulnificus* triggers an acute inflammatory response that occurs in two main phases. The early phase [3 h post infection (hpi)] is characterized by upregulation of proinflammatory cytokines genes, antiviral cytokines, and genes for antiviral factors. The late phase (12 hpi) is characterized by upregulation of typical inflammatory cytokines and RNA-based immune response. Both RBCs and WBCs were found to be transcriptionally active and contribute to this atypical immune response, especially in the short term. To summarize, the study confirmed the hypothesis that sepsis death of *V. vulnificus* hosts is due to an early cytokine storm triggered by the pathogen, and proposed a set of selected marker genes, including npsn, cox2, mmp9, and sitd1 for the early detection of fish septicemia caused by *V. vulnificus*.

By comparative genomics, phylogenomics and Phyre2, Chaurasia et al. discovered that the virulence determinant (VM) proteins in the virulent *Leptospira* spp. are diverse in copy numbers, but all possess a tandemly repeated ricin B-like lectin N-terminal domain and a carboxyl terminal toxin domain. *In vitro* and *ex vivo* assays with recombinant VM protein LA3490 (aka Q8F0K3) of *L. interrogans* serovar Lai and its N-terminal variant t3490 demonstrated for the first time that the ricin B-like domain has a binding specificity for terminal galactosyl residues and the *Leptospira* toxin has an endo- and exo-DNase activity in its carboxyl terminal domain. The results further suggest that the ricin B-like domain is likely responsible for host cell targeting and internalization, while the C-terminal region mediates intracellular trafficking and cytotoxicity.

In summary, these articles have demonstrated how the omics-based technologies have, and will continue, to provide rapid and often more complete insights on zoonotic diseases from the biology and virulence attributes of the different pathogens to the pathogenesis including pathogen-host interaction and host responses, and allow identification of potential targets for development of vaccines and/or therapeutic drugs.

## Author contributions

All authors listed have made a substantial, direct, and intellectual contribution to the work and approved it for publication.

## Conflict of interest

The authors declare that the research was conducted in the absence of any commercial or financial relationships that could be construed as a potential conflict of interest.

## Publisher's note

All claims expressed in this article are solely those of the authors and do not necessarily represent those of their affiliated organizations, or those of the publisher, the editors and the reviewers. Any product that may be evaluated in this article, or claim that may be made by its manufacturer, is not guaranteed or endorsed by the publisher.

